# Warm-hearted businessmen, competitive housewives? Effects of gender-fair language on adolescents’ perceptions of occupations

**DOI:** 10.3389/fpsyg.2015.01437

**Published:** 2015-09-23

**Authors:** Dries Vervecken, Pascal M. Gygax, Ute Gabriel, Matthias Guillod, Bettina Hannover

**Affiliations:** ^1^Karel de Grote University College, AntwerpBelgium; ^2^University of Fribourg, FribourgSwitzerland; ^3^Norwegian University of Science and Technology, TrondheimNorway; ^4^Free University of Berlin, BerlinGermany

**Keywords:** gender stereotypes, gender-fair language, adolescence, stereotype content, stereotype change

## Abstract

Recent studies from countries with grammatical gender languages (e.g., French) found both children and adults to more frequently think of female jobholders and to consider women’s success in male dominated occupations more likely when the jobs were described in pair forms (i.e., by explicit reference to male and female jobholders, e.g., *inventeuses et inventeurs*; French feminine and masculine plural forms for *inventors*), rather than masculine only forms (e.g., *inventors*). To gain a better understanding of this phenomenon, we systematically varied the gender connotation of occupations (males overrepresented, females overrepresented, equal share of males and females) and measured additional dependent variables, predicting that gender fair language would reduce the impact of the gender connotation on participants’ perceptions. In a sample of 222 adolescents (aged 12–17) from French speaking Switzerland, we found that pair forms attenuated the difference in the ascription of success to male and female jobholders in gendered occupations and attenuated the differential ascription of warmth to prototypical jobholders in male vs. female dominated jobs. However, no effect of language form on the ascription of competence was found. These findings suggest that language policies are an effective tool to impact gendered perceptions, however, they also hint at competence-related gender stereotypes being in decline.

## Introduction

In recent years, the use of so-called gender-fair language has been strongly promoted. This language reform reflects the assumption that language, here gender-fair language, is a tool to influence people’s gendered perception of reality. For example, with respect to occupations, studies with adults and primary school children from countries with grammatical gender languages (e.g., French, German, Dutch, Spanish, Italian) suggest that they are perceived in a less gender-typed manner when they are described in gender-fair language, more specifically in pair forms (i.e., by explicit reference to both male and female jobholders, e.g., *inventeuses et inventeurs*; French feminine and masculine plural forms for inventors), rather than masculine plural forms (e.g., inventors; Braun et al., 1998; Heise, 2000, 2003; Stahlberg and Sczesny, 2001; Stahlberg et al., 2001; Rothmund and Scheele, 2004; Vervecken et al., 2013; Vervecken and Hannover, 2015; for a discussion of this issue for natural gender languages e.g., English; see, e.g., Gabriel et al., 2008; Garnham et al., 2012; Lassonde and O’Brien, 2013).

For example, in an experiment by Stahlberg and Sczesny (2001, Study 1), adult German participants were asked to write down the name of their favorite musicians or athletes. Participants received these instructions with either a masculine only form (*Musiker* [male or generic musician], *Sportler* [male or generic athlete]) or a pair form (Musikerin/Musiker [female/male musician]; Sportlerin/Sportler [female/male athlete). Results showed that participants who had received the role nouns in a pair form listed more female personalities then participants in the masculine only condition. Similar results have been reported with German and Dutch speaking Belgian primary school children as young as 6 years of age (Vervecken et al., 2013). Vervecken et al. (2013) (Studies 2 and 3) investigated primary school children’s perceptions of females’ and males’ success (i.e., who can succeed?) in traditionally male occupations. The occupational titles were presented to the children either in a masculine only form or a pair form. After being presented with an occupational title, children were asked “*who can succeed in this occupation*” and to indicate their response on a five-point scale (ranging from *1* = *only men* to *5* = *only women*): children in the pair form condition systematically perceived females’ and males’ success more equally than children in the masculine only form condition who attributed success predominantly to males.

To gain a better understanding of the above described phenomena, in this study, we wanted to investigate more systematically how language forms interact with the gender connotation of an occupation.

### Proportions of Males and Females Working in an Occupation Shape Gendered Perceptions of Prototypical Job Holders

Social role theory predicts that people make inferences about social groups from their typical social roles, for example occupational roles (e.g., Eagly and Koenig, 2014). Applied to gender, professions in which either males or females are clearly overrepresented will be the ones from which perceivers infer gender stereotypes (e.g., attributes which females or males supposedly have): women are traditionally seen as ‘communal’ (warm), e.g., nurturing or well-intended based on the social roles they are more likely to perform than men (e.g., nurse). By the same token, men are perceived as ‘agentic’ (competent), e.g., competitive or efficient as a result of the social roles which they more often have than women (e.g., manager; see Diekman and Eagly, 2000).

Empirical support comes from research showing a correspondence between the proportion of males and females working in an occupation and the ascription of gendered attributes to the prototypical job holder (e.g., Cejka and Eagly, 1999; Crawley, 2014; Eagly and Koenig, 2014): to the extent that an occupational group is perceived as dominated by women (e.g., childminder), people tend to believe that feminine qualities are required to be successful within these occupations (e.g., warm-hearted). When an occupational group is perceived as dominated by men (e.g., stock broker), people tend to believe that masculine qualities are essential for workers to be successful (e.g., competitive). Further support comes from experimental studies manipulating the distribution of males and females in occupations. For example Crawley (2014) varied the percentages of women and men who allegedly worked in different occupations. Participants were more likely to indicate that a university degree was needed if the occupation was, supposedly, primarily occupied by men than if the job was described as dominated by women.

When a social role, like an occupation, is described in a linguistic pair form (e.g., businesswomen and businessmen, housemen and housewives), explicit reference is made to both males and females. Considering the findings described above, we speculated that if it is a profession in which one gender is overrepresented, people should be less inclined to ascribe the characteristics of that occupation to the respective gender group, as the pair form makes them think of both genders when describing the prototypical job holder. For example, descriptions of an occupational group from a male dominated field in a masculine only form, like “businessmen,” will most likely trigger associations with stereotypically male, i.e., agentic traits: “businessmen are competent and self-confident people.” Describing the same occupational group in a pair form, like “businesswomen and businessmen,” may additionally trigger associations with stereotypically female, i.e., communal traits, such as “helpful” and/or “friendly.”

### Warmth and Competence as Core Dimensions of Gendered Perceptions of Prototypical Job Holders

To investigate whether the ascription of gendered attributes to prototypical job holders is influenced by the linguistic form (pair vs. masculine only) we used the dimensions of *warmth* and *competence*. According to the Stereotype Content Model (SCM, Fiske et al., 2002, 2007; Fiske, 2011), warmth and competence are two universal dimensions that guide people’s perception of others. Specifically, these two dimensions are driven by the need to evaluate whether others (a) have beneficial intentions for oneself and for one’s group (i.e., warmth dimension) and (b) have the ability to implement their good/bad intentions (i.e., competence dimension). Although women in general often receive more positive evaluations, women occupying more traditional roles (e.g., housewives) are perceived as warm but incompetent and those in non-traditional roles (i.e., businesswomen) as cold yet competent. We suggest that describing an occupational group in its masculine form only, like “successful businessmen,” may result in ascriptions of coldness and competence: although they are perceived as having bad intentions (e.g., people who sell something solely for personal gain), they are still perceived as competent (e.g., as very good at making money for themselves). Describing the same occupational group in a pair form (“businesswomen and businessmen”) should result in the ascription of comparably more warmth (e.g., people who want to sell useful things) and less competence (e.g., people who, after all, do not earn more money than people in other domains).

Indirect support for the assumption that gender-fair language might affect perceptions of warmth and competence comes from research comparing the impact of the masculine singular vs. the feminine singular form for job titles on evaluations of those jobs and their suitability for female applicants (Merkel et al., 2012; Formanowicz et al., 2013; Budziszewska et al., 2014). For example Formanowicz et al. (2013) demonstrated with both invented (Studies 1–2) and existing (Study 3) job titles that female applicants described with a feminine job title were evaluated as being less competent than applicants described with a masculine job title. While job titles in the feminine form lead to some devaluation of competence, women are often evaluated more favorably than men on warmth (cf. the “women are wonderful effect,” Eagly and Mladinic, 1994). Merkel et al. (2012) illustrated that the “women are wonderful-effect” can be induced by the linguistic form used to describe a professional: female professionals described with a feminine title (e.g., *avvocata* [female lawyer]) were judged as warmer than professionals described by a masculine title (e.g., *avvocato* [male lawyer]). Although these studies illustrate that male vs. female job titles trigger different perceptions of female professionals, no research has explored whether pair forms vs. masculine only forms have a different impact on gendered perceptions of an occupational group in general (i.e., prototypical female and male workers in a given occupation).

### Adolescence as Crucial Stage in Vocational Development

We will test our hypotheses with adolescents aged 12–17. Existing research on language effects was primarily done with children, focusing on the emergence of gendered linguistic concepts in the primary school years (e.g., Hyde, 1984; Schau and Scott, 1984; Vervecken et al., 2013; Vervecken and Hannover, 2015), or with adults, focusing on the practical importance of the use of different linguistic forms in everyday life, such as in job advertisements (see Stahlberg et al., 2007 for an overview). In contrast, research with adolescents is almost non-existent (see Chatard et al., 2005 for a noticeable exception). This is an unsatisfactory situation, as the transition from adolescence to adulthood is an important stage in vocational development, in which the gendered perception of occupations can play an essential role (Gottfredson, 2005; Lerner and Steinberg, 2009). Adolescence is a crucial stage in vocational development as youngsters get more realistic about their future career options and start to abandon unrealistic aspirations (Helwig, 2001; Blanchard and Lichtenberg, 2003; Hartung et al., 2005). However, perceptions of what is required to pursue different professions are often biased by gender stereotypes (e.g., Crawley, 2014), which are a result of associating occupations with one of the two genders (White and White, 2006; Eagly and Koenig, 2014).

While not directly investigated in this study, describing potential future professions to adolescents in gender-fair language may help to reduce the restrictions that boys, and more particularly girls, impose on themselves when deciding which occupations to aspire to.

### Research Hypotheses

In sum, the present study seeks to investigate the impact of linguistic forms (pair forms compared to masculine only forms) used to describe occupations in which either males are overrepresented, females are overrepresented (male or female gendered occupations), or in which males and females are represented about equally (gender-neutral occupation), on adolescents’ perceptions of these occupations. More specifically, we wanted to replicate the finding of previous studies conducted with children or adults, which show that linguistic forms impact the perception of the extent to which women and men can succeed in these occupations. In addition, we wanted to investigate the effect of linguistic forms on the ascription of warmth and competence to people performing these occupations. Against the background of the above described findings, we speculated that when presented with a profession in which one gender is overrepresented, people should be less inclined to ascribe the characteristics of that occupation (warmth, competence) to the respective gender group, as the pair form makes them think of both genders when describing the prototypical job holder.

Our hypotheses were as follows:

The use of pair forms (compared to masculine only forms) to describe occupations:

(1) will attenuate the difference in the ascription of success to males and females in gendered occupations (i.e., the deviation from the midpoint of the answering scale, indicating that males and females alike can succeed in the job, should be smaller);
(2) will attenuate the influence of the distribution of males and females in that occupation on the differential ascription of warmth to prototypical job holders (i.e., the difference in the ascription of warmth to holders of female vs. male jobs should become smaller);
(3) will attenuate the influence of the distribution of males and females in that occupation on the differential ascription of competence (i.e., the difference in the ascription of competence to holders of female vs. male jobs should become smaller).

## Materials and Methods

### Participants

Two hundred and twenty-two (*N* = 222) French-speaking adolescents from two different schools in Porrentruy (French speaking part of Switzerland) took part in this experiment (mean age = 14; range = 12–17; 114 female, 107 male, one participant did not indicate his/her gender). One female participant was removed from the analyses as she did not follow the instructions. This study was approved by the Ethics Committee of the Department of Psychology (University of Fribourg) and carried out in accordance with their recommendations. All participants have granted informed consent.

### Materials and Procedure

Participants (in group sessions) were orally presented with fifteen occupations (i.e., five female stereotyped, five male stereotyped, and five gender-neutral; see **Table 1**). Occupations were presented one after another in a set random sequence, which was the same for all participants. Participants were instructed to rate each occupation in a booklet on a series of 15 dimensions (for a description of the rating method see below). The experimenter waited until all participants were finished with rating an occupation before going on to the next one.

**Table 1:** Occupational titles (pair forms in parenthesis) used in the Experiment.

Assumed gender distribution within occupations	French	English translations
Male dominated	Camionneurs (et camionneuses) Inventeurs (et inventeuses) Maçons (et maçonnes) Mécaniciens (et mécaniciennes) sur auto Informaticiens (et informaticiennes)	Male (and female) truck drivers Male (and female) inventors Male (and female) bricklayers Male (and female) car mechanics Male (and female) computer scientist
Female dominated	Infirmiers (et infirmières) Babysitters (et babysittrices) Nettoyeurs (et nettoyeuses) Esthéticiens (et esthéticiennes) Educateurs (et éducatrices) de la petite enfance	Male (and female) nurse Male (and female) babysitters Male (and female) cleaners Male (and female) beauticians Male (and female) preschool teacher
Approximately equally distributed	Ecrivains (et écrivaines) Chanteurs (et chanteuses) Pharmaciens (et pharmaciennes) Sportifs (et sportives) Musiciens (et musiciennes)	Male (and female) writers Male (and female) singers Male (and female) pharmacists Male (and female) athletes Male (and female) musicians

To manipulate the distributions of males and females in occupations, we selected 15 occupations (see **Table 1**) from a list of 126 role nouns which had been normed with respect to the representation of males and females (in %) in the respective occupational group (Gabriel et al., 2008; Irmen and Schumann, 2011). We used the cut-off value >70% men to define male dominated jobs, >70% women to define female dominated jobs, and both men and women <60% to define gender neutral occupations.

To manipulate linguistic form, for half of the participants (*N* = 117) the occupational titles were orally presented in the masculine only form; the other half (*N* = 105) received the same occupational titles in the pair form. Each job title was orally accompanied by a short description of the jobholder’s tasks and activities. These descriptions were identical in both conditions.

To measure ascriptions of gendered attributes to prototypical job holders in the different occupations, we used the six items referring to warmth (e.g., *friendly, well-intended*) and the six items referring to competence (e.g., *efficient, expert*) from the scale of Fiske et al. (2002). Immediately after an occupation had been presented, participants were asked to indicate on five-point Likert scales (1 = *not at all*, 5 = *extremely*) how competent (e.g., efficient, expert) and warm (e.g., friendly, well-intended) they thought prototypical job holders would be. Cronbach’s alpha values were: for competence regarding female dominated occupations (α = 0.93), male dominated occupations (α = 0.87), and gender-neutral occupations (α = 0.90). Cronbach’s alpha values were: for warmth regarding female dominated occupations (α = 0.91), male dominated occupations (α = 0.93) and gender-neutral occupations (α = 0.93).

Finally, to measure perceptions of male and female success in the different occupations, we asked participants to indicate on a five-point Likert scale who they thought would succeed in each occupation (1 = *only men*, 3 = *men and women alike*, 5 = *only women*, Cronbach’s alpha values were: for female dominated occupations: α = 0.71; male dominated occupations α = 0.73; gender-neutral occupations α = 0.34).

For each dependent variable, means were calculated separately for female dominated, male dominated, and gender-neutral occupations^1^. All rating scales were labeled numerically and presented with equidistant markings to ensure that the scales were considered as continuous, hence reliable (Krosnick and Berent, 1993). Data were analyzed using parametric statistics as each subscale consisted of at least five items (Boone and Boone, 2012). In some cases (i.e., ascriptions of success), normality tests (i.e., Kolmogorov–Smirnov) indicated non-normal distributions. In these cases, we ran additional non-parametric statistics. As our sample size was relatively large (all *n* > 30; Hays, 1994), and as there was no difference between the two statistics (unless otherwise stated), we only present the results from the parametric statistics.

## Results

### Differential Ascription of Success to Male and Female Jobholders in Gendered Occupations

To test our first hypothesis that language forms would impact the ascription of success to men and women, we conducted a 2 (*Form*: Pair form vs. Masculine only) × 2 (*Gender of respondent*: Female vs. Male) × 3 (*Assumed gender distribution within occupations*: Female vs. Male vs. Neutral) factorial mixed ANOVA on the *Perceived success of men and women*, with *Age* as a covariate, *Form* and *Gender of respondent* as between-participant factors and *Assumed gender distribution* as a within-participant factor.

Results showed a main effect of Assumed gender distribution, *F*(2,430) = 4.07, *p* < 0.05, η = 0.02, suggesting that for female dominated jobs, success was considered more likely for women than for men (*M* = 3.56, *SE* = 0.03). In contrast, men were perceived to more likely succeed in male dominated jobs than women (*M* = 2.24, *SE* = 0.03, *p* < 0.001). As expected, for gender-neutral occupations, i.e., a job in which the genders are represented about equally, participants’ mean response reflected the midpoint of the answering scale: women were considered as likely to succeed as men (*M* = 2.99, *SE* = 0.01, pairwise LSD comparison at *p* < 0.001).

In support of hypothesis 1, the analysis revealed an interaction between *Assumed gender distribution* and *Form*: *F*(2,430) = 12.73, *p* < 0.001, η^2^ = 0.06, indicating that compared to the masculine only condition, in the pair form condition participants’ mean responses were closer to the midpoint of the answering scale (*3 = women and men can succeed equally*). When occupations were presented in pair form, rather than the masculine only form, the perception that women and men can equally succeed in occupations increased for male dominated occupations [Masculine Form: *M* = 2.11, *SE* = 0.04; Pair Form: *M* = 2.38, *SE* = 0.04, *t*(219) = -4.51; *p* < 0.05] and female dominated occupations [Masculine Form: *M* = 3.63, *SE* = 0.04; Pair Form: *M* = 3.48, *SE* = 0.05, *t*(219) = 2.46; *p* < 0.05^2^]. Hence, the deviation from the midpoint of the answering scale (indicating differential ascription of success to men and women) was more pronounced in the masculine only condition [Male dominated occupations: *M* = 3.63, *SE* = 0.03; Female dominated occupations: *M* = 2.11, *SE* = 0.04, *t*(116) = 25.03; *p* < 0.001; Cohen’s *d* = 2.32] than in the pair form condition [Male dominated occupations: *M* = 3.48, *SE* = 0.06; Female dominated occupations: *M* = 2.38, *SE* = 0.05, *t*(104) = 11.74; *p* < 0.05; Cohen’s *d* = 1.14]. Interestingly, perceived success of women and men in gender neutral occupations, i.e., jobs in which the genders are represented about equally was also influenced by the linguistic form: [Masculine Form: *M* = 2.96, *SE* = 0.02; Pair Form: *M* = 3.04, *SE* = 0.02, *t*(219) = 3.31; *p* < 0.05]. Neither *Gender of respondent* nor *Age* were significant predictors and none of the other interaction terms with *Form* were statistically significant.

**FIGURE 1:**
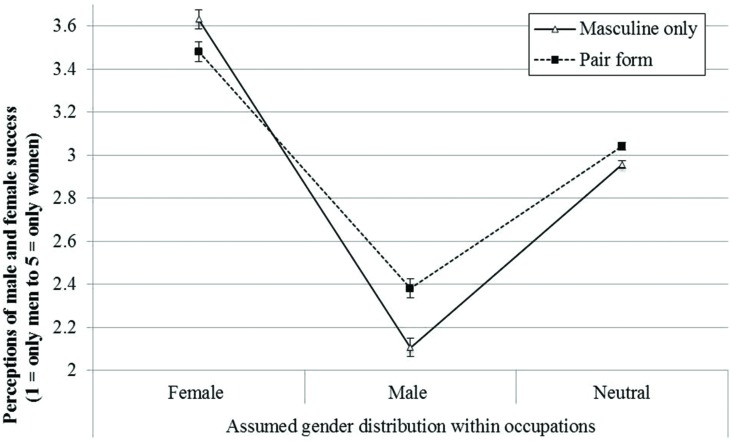
**Mean perceptions of success for men and women in occupations with different gender distributions (scale from 1 = only men to 5 = only women)**.

In summary, as expected, adolescents of all ages and regardless of their gender, perceived success in gendered occupations to be more equally shared by women and men when the job had been described to them in a pair form rather than in the masculine form only (see **Figure 1**).

### Ascriptions of Warmth and Competence to Jobholders in Male and Female Dominated and in Gender-Neutral Occupations

To test our hypotheses that language form used to describe occupational titles would influence the ascription of warmth and competence to prototypical jobholders, we conducted separate analyses for warmth and competence in line with previous research (e.g., Vervecken and Hannover, 2012; Budziszewska et al., 2014).

#### Warmth

We performed a 2 (*Form*: Pair form vs. Masculine only) × 2 (*Gender of respondent*: Female vs. Male) × 3 (*Assumed gender distribution*: Female vs. Male vs. Neutral) factorial mixed ANOVA on *warmth*, with *Age* as a covariate, *Form* and *Gender of respondent* as between-participant factors and *Assumed gender distribution* as a within-participant factor.

In support of hypothesis 2, the analysis revealed a statistical interaction between *Assumed gender distribution* and *Form*, *F*(2,430) = 3.71, *p* < 0.05, η^2^ = 0.03. When occupations were presented in pair form, ascriptions of warmth increased for male dominated occupations (Masculine Form: *M* = 3.24, *SE* = 0.06; Pair Form: *M* = 3.29, *SE* = 0.06) but decreased for female occupations (Masculine Form: *M* = 3.84, *SE* = 0.04; Pair Form: *M* = 3.78, *SE* = 0.05). Hence, warmth ascribed to prototypical jobholders differed more strongly between male dominated vs. female dominated jobs when the job had been presented in the masculine only form [difference of 0.59, *SE* = 0.04, *t*(116) = 13.54, *p* < 0.01, Cohen’s *d* = 1.25], compared to when the occupation had been described in pair forms [difference of 0.51, *SE* = 0.05, *t*(103) = 10.20, *p* < 0.01, Cohen’s *d* = 1.00]. Also, the difference in warmth ascribed to holders of gender-neutral occupations (Masculine Form: *M* = 3.40, *SE* = 0.05; Pair Form: *M* = 3.51, *SE* = 0.05) vs. female dominated occupations decreased when the job had been described in a pair form [difference of 0.28, *SE* = 0.04, *t*(103) = 6.51, *p* < 0.01, Cohen’s *d* = 0.63] compared to when it had been presented in the masculine only form [difference of 0.44, *SE* = 0.04, *t*(116) = 12.08, *p* < 0.01, Cohen’s *d* = 1.12].

The analysis also revealed a significant statistical interaction between *Assumed gender distribution* and *Gender of the respondent*, *F*(2,430) = 5.89, *p* < 0.01, η^2^ = 0.03, suggesting that girls and boys differed in their attributions of warmth when considering female dominated job [Girls: *M* = 3.89, *SE* = 0.04; Boys: *M* = 3.71, *SE* = 0.05, *t*(220) = 2.74, *p* < 0.01] but not when considering male dominated [Girls: *M* = 3.26, *SE* = 0.05; Boys: *M* = 3.27, *SE* = 0.06, *t*(219) < 1, *ns*], or gender-neutral occupations [Girls: *M* = 3.48, *SE* = 0.05; Boys: *M* = 3.41, *SE* = 0.05, *t*(219) < 1, *ns*]. *Age* was not a significant predictor for adolescents’ warmth related attributions toward occupations.

**FIGURE 2:**
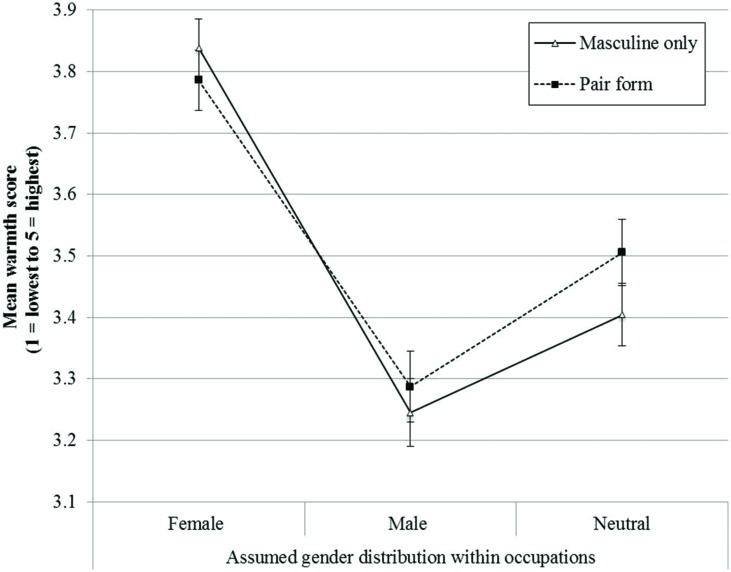
**Mean scores on the *warmth* dimension for occupations with different gender distributions**.

In summary, when occupations were presented in a pair form rather than in the masculine form only, the differential ascription of warmth to prototypical job holders of male dominated, female dominated, and gender-neutral occupations was attenuated, regardless of participants’ age and gender. As apparent in **Figure 2**, the effect of linguistic form on warmth-related attributions mirrors the pattern of linguistic form on gendered representations of women’s and men’s success.

#### Competence

To test research hypothesis 3, we again conducted a 2 (*Form*: Pair form vs. Masculine only) × 2 (*Gender of respondent*: Female vs. Male) × 3 (*Assumed gender distribution within occupations*: Female vs. Male vs. Neutral) factorial mixed ANOVA on *competence*, with *Age* as a covariate, *Form* and *Gender of respondent* as between-participant factors and *Assumed gender distribution* as a within-participant factor.

There was only a main effect of the *Gender of the respondent*, *F*(1,216) = 5.89, *p* < 0.05, η^2^ = 0.03, showing that girls in general ascribed higher levels of competence (*M* = 3.79, *SE* = 0.04) than boys (*M* = 3.64, *SE* = 0.06). There were no other significant main or interaction effects (all *p* > 0.20). Hence, hypothesis 3 was not supported.

## Discussion

By combining work on the impact of gender-fair language on mental representations (e.g., Stahlberg et al., 2007) with work on stereotypes (Fiske et al., 2002; Fiske, 2011; Eagly and Koenig, 2014) the present study investigated how different linguistic forms (i.e., pair form vs. masculine only form) used to present female dominated, male dominated, and gender-neutral jobs impact adolescents’ gendered perceptions, in particular their ascriptions of success, warmth and competence to male and female job holders.

### Gendered Perceptions of Occupational Success

In our sample of 222 adolescents (aged 12–17) from French speaking Switzerland we found, in line with our first hypothesis, that regardless of whether males, females or neither gender was overrepresented in an occupation, presentation in the linguistic pair form, rather than the masculine form only, triggered more gender-balanced representations of occupational success. These results broaden the findings of previous studies which typically only investigated male dominated occupations (for a review, see, Stahlberg et al., 2007). To more systematically investigate how language forms interact with the gender connotation of occupations, in our study we not only presented male dominated, but also female dominated and gender-neutral jobs. Interestingly, not only in male stereotyped jobs but also in female and gender-neutral jobs, occupational success was more evenly attributed to males and females when the occupation was described in a linguistic pair form, rather than the masculine generic form only.

These results suggest that subtle linguistic markers, besides other factors potentially influencing gender stereotyping (for a review, see Blakemore et al., 2009), can have an impact on the extent to which adolescents think that women and men can be professionals in the same domains. It seems that masculine only forms vs. pair forms operate like primes, increasing the mental accessibility of either male job holders or, respectively, female job holders. The linguistic markers activate the corresponding mental representations which in turn guide recipients’ categorization and interpretation of the information (cf. Bargh, 2014; Stupica and Cassidy, 2014). Our findings suggest that such “natural priming effects” (Bargh, 2014, p. 218) are important in everyday life, as they influence the perception of females’ and males’ occupational success.

### Gendered Ascriptions of Warmth and Competence

Going beyond the scope of previous studies, we not only looked at the effects of linguistic forms on the perception of males’ and females’ occupational success, but also on the ascription of warmth and competence to prototypical jobholders. We assumed that linguistic pair forms make participants think of both genders, such that the impact of an unequal gender distribution within an occupation on their perceptions of the jobs would be attenuated. As a result, ascriptions of warmth and competence, as two universal dimensions guiding people’s perceptions of others (Fiske et al., 2002), should differ less between holders of female dominated vs. male dominated occupations when the jobs are described in a pair form, rather than the masculine form only. Results confirmed our expectation that the difference in the ascription of warmth to holders of female dominated vs. male dominated occupations was smaller in the pair form condition (compared to the masculine only condition). Furthermore, the difference in the ascription of warmth to holders of female dominated vs. gender neutral occupations was also smaller in the pair form condition (compared to the masculine only condition). It seems that when a male dominated occupation (e.g., businessmen) is presented in a pair form, adolescents are inclined to attribute more warmth to the prototypical job holder. However, when a female dominated occupation (e.g., child care taker) is described in pair form, stronger associations with coldness are triggered.

The ascription of warmth being influenced by our experimental manipulation is in line with the results of Merkel et al. (2012), who found that female targets whose job had been described in feminine forms were perceived as warmer than female targets whose job had been described in a masculine only form. Our findings complement the ones reported by Merkel et al. (2012) in that we could show that pair form use influenced attributions of warmth in general (i.e., to female and male workers in a certain occupation), and furthermore, that ascriptions of warmth actually decreased when female dominated occupations had been described in pair forms.

While the stronger ascription of warmth to job holders in male dominated and the weaker ascription of warmth to job holders in female dominated jobs is consistent with our hypotheses, unexpectedly, competence ratings were unaffected by our linguistic manipulation. Interestingly, Merkel et al. (2012) also found that *competence* ratings remained unaffected by the linguistic form in which females’ occupations had been presented. In fact, some authors (e.g., Wojciszke et al., 1998; Fiske et al., 2007) have argued that perception of warmth is primary to ascriptions of competence, with others’ intentions being more prominent – in an evolutionary perspective – than others’ abilities to act on those intentions. From our results, one could then argue that linguistic forms are used to make inferences on moral and social dimensions but not on a person’s competency or expertise.

Alternatively, our linguistic manipulation not having an impact on the ascription of competence can hint at competence-related gender stereotypes being in decline. A recent study investigating implicit stereotypes about women in Germany did not replicate the women-incompetence stereotype (Ebert et al., 2014) as described by the SCM (Fiske et al., 2002; for similar findings for Spain see López-Sáez et al., 2008). While such a change in the female stereotype has been demonstrated only in some cultural contexts, poll data from US national surveys point in a similar direction (Newport, 2001; Pew Research Center, 2008): they show that nowadays women are increasingly perceived as more intelligent than men in the general population (see Wood and Eagly, 2012, for a review). In terms of social role theory, our finding that although linguistic form had an impact on perceptions of women and men’s success it did not affect competence-related evaluations, could indicate a shift in gender stereotyping: since women are no longer associated with lower competence, differences in the percentage of women in an occupation or variations in the mental accessibility of female job holders – as caused by our linguistic manipulation – can no longer be expected to have an impact on competence perceptions of prototypical job holders.

### Practical Relevance of Our Findings

The findings from the current experiment demonstrate that adolescents are sensitive to gender information in occupational titles and use this information to make gendered inferences about the occupations. It seems that the generic use of masculine only forms when describing occupations is likely to lead adolescents to restrictive, gender exclusive associations and perceptions about occupations. This is an especially important finding as the transition from adolescence to adulthood is an important stage in vocational development in which gender stereotyped perceptions of occupations play an essential role (Gottfredson, 2005; Lerner and Steinberg, 2009).

While changing occupational gender stereotypes has long been recognized as a key for closing the occupational gender gap, few interventions have been investigated to tackle this issue in adolescence. This is especially dissatisfying as adolescents’ career aspirations are important predictors for educational and occupational status in adulthood (Sewell and Hauser, 1972; Campbell, 1983; Kao and Thompson, 2003; Feliciano and Rumbaut, 2005; Beal and Crockett, 2010; Lee et al., 2012). For example Beal and Crockett (2010) found in a longitudinal study that adolescents’ educational expectations were positively associated with educational attainment in young adulthood. In a similar vein, Sewell and Hauser (1972) demonstrated that post-secondary educational attainment at age 25 was significantly predicted by aspirations students held in adolescence, and educational attainment, in turn, positively predicted earnings at the age of 28.

Any intervention that aims to alter aspects of representational biases may well-contribute to reducing occupational gender segregation (Weisgram et al., 2011; Eagly and Koenig, 2014; Liben and Coyle, 2014). Gender-fair language use by teachers, parents, or the media may thus contribute to an attenuation of adolescents’ gender related stereotypes about occupations.

Our findings are also consistent with the view that the current extensive use of the masculine only form (Blaubergs, 1980; Parks and Robertson, 1998; Bußmann and Hellinger, 2003; Mucchi-Faina, 2005; Koeser and Sczesny, 2014; Kuhn and Gabriel, 2014) may well-contribute to shaping, or at least maintaining, gender stereotypes. Consequently, enforcing or encouraging the use of pair forms in grammatical gender languages when referring to mixed gender groups or to groups whose gender composition is unknown or irrelevant seems to be an effective strategy to counter gender stereotypes. Our findings substantiate the effectiveness of recent linguistic reforms as currently promoted by many professional organizations, publishing companies, and governmental organizations (e.g., Duden, 2006; European Commission, 2008; American Psychological Association, 2009): they advocate gender-fair language use and reject the notion that the masculine form can be generic. Unfortunately, these language reforms contrast with the still common use of the masculine only form in various applied settings, for example, in schools, as illustrated by studies on teachers’ language practice (e.g., Vervecken et al., 2010) or schoolbooks’ contents (e.g., Moser and Hannover, 2014).

It is possible that pair forms might promote wider interest in traditionally constrained disciplines such as the STEM fields (i.e., Science, Technology, Engineering, and Mathematics). In fact, a recent review by Liben and Coyle (2014) suggests that one tangible way to promote interest in STEM fields might be to alter the traditionally masculine image of these occupations to a more feminine one.

### Limitations of Our Study and Future Directions

In this article, we argued that the use of gender fair language to describe occupations has an impact on adolescents’ perceptions of occupations. Although our evidence is quite compelling, the generalizability of our findings could be discussed.

First, in the present study, we had to restrict the experimental stimulus material to fifteen role nouns. Therefore, we cannot provide by-items analyses, and the generalizability of our findings to other occupations remains to be tested in future studies. However, according to the theory of generalizability (Cronbach et al., 1963), Cronbach’s alpha can be viewed as a measure of how well the *sum score* on the selected items captures the expected score in the entire domain, even if that domain is heterogeneous. Hence, the very high Cronbach’s alpha values for warmth and competence ratings across the five occupations of each of the three groups of occupations suggest that our findings may be, in fact, generalized to other occupations.

Second, we generally clustered all male, female and gender-neutral occupations together. However, other categorizations and extra subdivisions within occupations are conceivable. For example, one could order occupations using the RIASEC model (cf. Holland, 1997), based on stereotypical personality types (Realistic, Investigative, Artistic, Social, Enterprising, and Conventional), or categorize them according to whether they belong to Science, Technology, Engineering and Mathematics (STEM-fields). Using a broader range of occupations and dividing them into meaningful subcategories could provide a more detailed insight into the effects of gender fair language.

Third, whereas the results of the present cross-sectional experiment illustrate effects of gender fair language shortly after it is presented, it is difficult to make inferences about long-term effects. A full account of the impact of gender fair language on adolescents’ development of occupational gender stereotypes and their subsequent educational and vocational development could only be provided by longitudinal study designs. Although there is some evidence suggesting that repetitively combining role nouns with the non-stereotypical gender (e.g., surgeon/mother) indeed may have longer-term impact (e.g., Finnegan et al., 2015), longitudinal research on gender fair language does not exist at this time. Future research may want to explore this. For example, some teachers could be trained in using gender fair language. Simultaneously the development of gender-role beliefs in their students could be monitored over a longer period of time and compared to students whose teachers use traditional language (masculine only forms). Similarly, textbooks using either gender fair or traditional language could be randomly assigned to different school classes. Again, the development of adolescents’ gender-role beliefs could be monitored and compared.

## Conflict of Interest Statement

The authors declare that the research was conducted in the absence of any commercial or financial relationships that could be construed as a potential conflict of interest.

## Footnotes

1 Note that, for each job, we also added a self-efficacy question (i.e., “*Imagine you wanted to become [job], how confident are you that you would pass the qualification test required to do this job?*,” 1 = *not confident at all* to 5 = *extremely confident*), to mimic Chatard et al. (2005) and Vervecken and Hannover (2015). Although we did find, as in previous studies, that girls felt more confident about female stereotyped jobs, and boys more confident about male stereotyped jobs, contrary to what Chatard et al. (2005) had found, our experimental manipulation had no effect on adolescents’ self-efficacy beliefs.

2 When running a non-parametric statistic, this effect was not significant, *U*(220) = 5597, *Z* = -1.05, *p* > 0.05.
